# Diagnosing Periprosthetic Joint Infection: Has the Era of the Biomarker Arrived?

**DOI:** 10.1007/s11999-014-3543-8

**Published:** 2014-03-04

**Authors:** Carl Deirmengian, Keith Kardos, Patrick Kilmartin, Alexander Cameron, Kevin Schiller, Javad Parvizi

**Affiliations:** 1 CD Diagnostics Inc, Lankenau Institute for Medical Research, 100 Lancaster Avenue, MOB 456, Wynnewood, PA 19096 USA; 2 The Rothman Institute, Thomas Jefferson University, Philadelphia, PA USA

## Abstract

**Background:**

The diagnosis of periprosthetic joint infection (PJI) remains a serious clinical challenge. There is a pressing need for improved diagnostic testing methods; biomarkers offer one potentially promising approach.

**Questions/purposes:**

We evaluated the diagnostic characteristics of 16 promising synovial fluid biomarkers for the diagnosis of PJI.

**Methods:**

Synovial fluid was collected from 95 patients meeting the inclusion criteria of this prospective diagnostic study. All patients were being evaluated for a revision hip or knee arthroplasty, including patients with systemic inflammatory disease and those already receiving antibiotic treatment. The Musculoskeletal Infection Society (MSIS) definition was used to classify 29 PJIs and 66 aseptic joints. Synovial fluid samples were tested by immunoassay for 16 biomarkers optimized for use in synovial fluid. Sensitivity, specificity, and receiver operating characteristic curve analysis were performed to assess for diagnostic performance.

**Results:**

Five biomarkers, including human α-defensin 1-3, neutrophil elastase 2, bactericidal/permeability-increasing protein, neutrophil gelatinase-associated lipocalin, and lactoferrin, correctly predicted the MSIS classification of all patients in this study, with 100% sensitivity and specificity for the diagnosis of PJI. An additional eight biomarkers demonstrated excellent diagnostic strength, with an area under the curve of greater than 0.9.

**Conclusions:**

Synovial fluid biomarkers exhibit a high accuracy in diagnosing PJI, even when including patients with systemic inflammatory disease and those receiving antibiotic treatment. Considering that these biomarkers match the results of the more complex MSIS definition of PJI, we believe that synovial fluid biomarkers can be a valuable addition to the methods utilized for the diagnosis of infection.

**Level of Evidence:**

Level II, diagnostic study. See Instructions for Authors for a complete description of levels of evidence.

## Introduction

Periprosthetic joint infection (PJI) accounts for 25% of failed knee arthroplasties [6] and 15% of failed hip arthroplasties [7]. Concern regarding the predicted economic impact of PJI, due to the increasing national volume of joint arthroplasties [13, 24] and increasing rate of infection [25, 26], is well justified. In caring for a painful joint arthroplasty, the ability to distinguish between septic and aseptic failure of the prosthesis is critical, as the treatment for PJI necessitates unique surgical strategies that aim to eradicate the infecting organism(s) [15, 32, 40]. Currently, surgeons utilize a wide spectrum of tests in an attempt to diagnose PJI, including (1) local measures of synovial inflammation [3, 12, 18, 38] (synovial fluid white blood cell [WBC] count and differential, synovial tissue histology), (2) systemic measures of inflammation [1, 10, 16] (serum C-reactive protein [CRP] level, erythrocyte sedimentation rate [ESR], IL-6), (3) radiographic tests [21, 31, 36, 37] (radiographs, bone scan, MRI, CT, positron emission tomography), and (4) bacterial isolation techniques [2, 17, 27, 42] (Gram stain, culture). Facing the challenge of accurately diagnosing PJI, the Musculoskeletal Infection Society (MSIS) recently published a definition of PJI [33], utilizing a combination of clinical data and six of the above tests.

There is substantial evidence that there exists a primitive, but specific, innate immune response to pathogens [9, 14, 23, 28, 29, 39]. In fact, the recognition of pathogens by the innate immune system triggers a cascade of protective pathways in the host. Microarray techniques have demonstrated a unique gene expression signature exhibited by the synovial fluid WBCs from infected joints, characteristic of the innate host immune response to infection [9]. This unique response to infection was also confirmed at the level of the proteome, revealing several biomarkers that diagnostically outperformed the currently available tests for PJI [8, 22].

For more than 8 years, our group has been interested in the discovery and evaluation of biomarkers for PJI [8, 9, 22], and we have identified 16 biomarkers of interest. In this study, we evaluated the diagnostic characteristics of these 16 promising synovial fluid biomarkers for PJI.

## Patients and Methods

### Study Design

The study was approved by the institutional review board. As part of a biomarker screening program initiated in 2009, our institution archives and prospectively annotates synovial fluid samples from the patients of adult arthroplasty surgeons. Patient inclusion in the current study required (1) an evaluation for possible infection of a THA or TKA, (2) sufficiently annotated clinical and laboratory data for classification by the MSIS criteria for PJI, and (3) sufficient synovial fluid for study methods. We did not exclude from this study patients receiving antibiotics before aspirations or patients having the diagnosis of a systemic inflammatory disease. Patients aspirated within 4 weeks after an index procedure and patients with an adverse tissue reaction to metal debris were excluded, as the MSIS definition for PJI does not include specific considerations for these diagnoses.

We prospectively evaluated and classified patients with PJI as defined by the MSIS [33] (Table 1). Although the MSIS criteria were not specifically designed to rule out PJI and PJI is acknowledged to potentially exist without meeting the MSIS criteria, those patients not meeting the MSIS criteria for PJI were, by default, classified as aseptic. Additionally, sex, age, joint (hip/knee), surgical findings, and isolated organism were recorded when pertinent. Sample size could not be calculated with any statistical rigor given the novel biomarkers being evaluated. Therefore, we chose to study a population of patients larger than those in previously published studies [8, 19, 22] that have demonstrated the diagnostic value of synovial fluid biomarkers.

**Table 1:** MSIS Workgroup standard definition for PJI

One of the following must be met for diagnosis of PJI:
(1) A sinus tract communicating with the prosthesis
(2) A pathogen is isolated by culture from two separate tissue or fluid samples obtained from the affected prosthetic joint
(3) Four of the following six criteria exist:
(a) Elevated ESR and CRP (ESR > 30 mm/hour; CRP > 10 mg/L)
(b) Elevated synovial fluid WBC count (> 3000 cells/μL)
(c) Elevated synovial fluid neutrophil percentage (> 65%)
(d) Presence of purulence in the affected joint
(e) Isolation of a microorganism in one periprosthetic tissue or fluid culture
(f) > 5 neutrophils per high-powered field in 5 high-power fields observed from histologic analysis of periprosthetic tissue at ×400 magnification

MSIS = Musculoskeletal Infection Society; PJI = periprosthetic joint infection; ESR = erythrocyte sedimentation rate; CRP = C-reactive protein; WBC = white blood cell.

### Patients

Ninety-five patients met the criteria of the study; these patients had 66 arthroplasties believed to be aseptic failures and 29 arthroplasties diagnosed with PJI.

Patients classified as having an aseptic joint included 32 men and 34 women, with a mean age of 67 years (range, 41–86 years). This group included nine hip arthroplasties, 55 knee arthroplasties, and two knee cement spacers. The diagnoses included 51 patients with aseptic loosening, three patients with instability, two patients with bearing surface wear and well-fixed implants, and 10 patients with pain but no mechanical diagnosis. Eleven patients (17%) also had a diagnosis of systemic inflammatory disease, including rheumatoid arthritis (four), pseudogout (two), psoriasis (one), Crohn’s disease (one), sarcoidosis (one), polymyalgia rheumatica (one), and hepatitis C (one). Four patients (6%) were taking a medication that modulates the immune system at the time of the diagnostic aspiration. Three patients in the aseptic group had an isolated positive culture that was considered a false positive, as the MSIS minor criteria for PJI were not met. All three patients had one isolated culture growing *Staphylococcus epidermidis*, two in “broth only” and the third with “light growth” on solid medium. All other preoperative and intraoperative cultures from these patients (at least two additional for each patient) were negative. No antibiotic treatments were provided to these patients, as the surgeons considered the results to be false positives. None of these patients had any other positive MSIS minor criteria. Followup of 18 months, 4 months, and 4 months revealed no further surgeries for these patients.

Patients classified as having PJI included 12 men and 17 women, with a mean age of 66 years (range, 49–89 years). This group included two hip arthroplasties, 26 knee arthroplasties, and one knee cement spacer. Among the 29 patients diagnosed with PJI, 23 were culture positive and six were culture negative. Organisms isolated included methicillin-sensitive *Staphylococcus aureus* (six), methicillin-resistant *S. aureus* (four), *S. epidermidis* (seven), *Streptococcus mutans* (one), *Streptococcus sanguinis* (one), *Streptococcus gordonii* (one), *Corynebacterium striatum* (one), *Escherichia coli* (one), and *Serratia marcescens* (one). Eight patients with PJI (28%) also had a diagnosis of systemic inflammatory disease, including rheumatoid arthritis (three), chronic lymphocytic leukemia (one), myelodysplastic syndrome (one), multiple sclerosis (one), gout (one), and hepatitis C (one). Two patients (7%) were taking a medication that modulates the immune system at the time of the diagnostic aspiration. Six patients diagnosed with a PJI (21%) were being treated with antibiotics at the time of aspiration.

The relevant clinical and MSIS laboratory values for patients with PJI versus those with aseptic disease are shown (Table 2).

**Table 2:** MSIS relevant laboratory and clinical findings

Finding	Aseptic group (n = 66)	PJI group (n = 29)
Sinus (number of patients)	0	4
At least one positive culture (number of patients)	3	23
ESR (mm/hour)*	15 (11–20)	86 (71–107)
CRP (mg/L)*	4 (3–6)	122 (72–184)
SF WBC count (cells/μL)*	400 (300–655)	29,170 (10,755–47,000)
Neutrophil %*	13 (5–27)	89 (86–92)

* Values are expressed as median, with 95% CI in parentheses; MSIS = Musculoskeletal Infection Society; PJI = periprosthetic joint infection; ESR = erythrocyte sedimentation rate; CRP = C-reactive protein; SF = synovial fluid; WBC = white blood cell.

### Sample Preparation and Biomarker Analysis

Synovial fluid was delivered to the laboratory immediately after aspiration. Centrifugation was used to separate all particulate and cellular material from each synovial fluid sample, and the resulting supernatant was aliquoted and frozen at −80° C.

Based on a review of our previous studies on biomarkers for PJI [8, 9, 22], as well as the general literature on sepsis biomarkers, we chose to screen 43 biomarkers that could potentially be diagnostic for PJI (Table 3). These 43 biomarkers were screened with a small subset of representative aseptic and PJI samples to identify markers that demonstrated an elevation in the setting of PJI. The 16 biomarkers evaluated in this current study demonstrated the greatest and most consistent elevations in the screening process: human α-defensin 1–3 (α-defensin), IL-1α, IL-1, IL-6, IL-8, IL-10, IL-17, granulocyte colony-stimulating factor (G-CSF), vascular endothelial growth factor (VEGF), CRP, neutrophil elastase 2 (ELA-2), lactoferrin, neutrophil gelatinase-associated lipocalin (NGAL), resistin, thrombospondin, and bactericidal/permeability-increasing protein (BPI).

**Table 3:** Forty-three biomarkers initially screened for inclusion in this study

Proteins passing screen (n = 16)	Proteins failing screen (n = 27)
Human α-defensin 1-3	Procalcitonin
Interleukin 1α	Transforming growth factor α
Interleukin 1β	Cathelicidin (LL-37)
Interleukin 6	Lipopolysaccharide binding protein
Interleukin 8	Calcitonin gene-related peptide
Interleukin 10	Orsomucoid
Interleukin 17	Nibrin
Granulocyte colony-stimulating factor	Tumor necrosis factor-stimulated gene 6 protein
Vascular endothelial growth factor	Plekstrin
C-reactive protein	Superoxide dismutase 2
Neutrophil elastase 2	Urokinase
Lactoferrin	Migration inhibitory factor
Neutrophil gelatinase-associated lipocalin	Plasminogen activator inhibitor type 1
Resistin	Soluble Fas
Thrombospondin 1	Soluble Fas ligand
Bactericidal/permeability-increasing protein	Soluble intercellular adhesion molecule 1
	Soluble vascular cell adhesion molecule 1
	Granzyme B
	Heat shock protein 70
	Macrophage inflammatory protein 1α
	Macrophage inflammatory protein 1β
	Matrix metalloproteinase 8
	Tumor necrosis factor α
	Interferon-γ inducible protein
	Fibroblast growth factor 2
	α-2 macroglobulin
	Skin-derived antileukoprotease

All immunoassays were optimized by laboratory scientists with specific expertise in immunoassay development. Assays were optimized to achieve an appropriate dynamic range and minimize the sample matrix effect. Immunoassays for the following synovial fluid biomarkers were generated using reagents from EMD Millipore Corp (Billerica, MA, USA) and measured using the bead-based platform from Luminex Corp (Austin, TX, USA): IL-1α, IL-1, IL-6, IL-8, IL-10, IL-17, G-CSF, VEGF, ELA-2, lactoferrin, NGAL, resistin, and thrombospondin. Immunoassays for the following synovial fluid biomarkers were generated using reagents from Hycult Biotech (Uden, The Netherlands) and measured in duplicate by standard ELISA: CRP, BPI, and α-defensin.

### Data Analysis

The diagnostic performance of each test was assessed using the MSIS definition as the gold standard. The diagnostic value of each biomarker was evaluated by receiver operating characteristic (ROC) curve analyses. The sensitivity and specificity (and 95% CIs) of each synovial fluid biomarker were calculated at various thresholds for a correct test result. Test sensitivity was plotted against 1 − specificity for every tested threshold and the area under the curve (AUC) was calculated. A test with an AUC value of greater than 0.9 is considered to have excellent diagnostic strength, whereas an AUC of 0.5 indicates a test with no diagnostic strength. Optimum cutoff values for correspondence with the MSIS-defined diagnosis were determined by Youden’s J statistic. For purposes of ROC analysis, raw data were processed according to the following rules: (1) the lowest reportable value was used for any samples that had a concentration below the limit of detection for an assay and (2) samples with results above the measuring range of an assay were diluted into range and corrected for dilution.

For samples with the diagnosis of infection, we compared the concentrations of select biomarkers to the synovial fluid WBC count and to each other by Pearson correlation. The following descriptions were utilized: r > 0.6 = strong positive relationship, +0.40 < r < +0.59 = moderate positive relationship, +0.19 < r < +0.39 = weak positive relationship, +0.20 > r > −0.19 = no relationship, −0.20 > r > −0.39 = weak negative relationship, −0.40 > r > −0.59 = moderate negative relationship, and r < −0.60 = strong negative relationship.

For all statistical analyses, we used GraphPad Prism^®^ software (Version 6; GraphPad Software Inc, San Diego, CA, USA).

## Results

Five biomarkers (α-defensin, ELA-2, BPI, NGAL, and lactoferrin) correctly predicted the diagnosis as defined by the MSIS criteria for every patient in the study. These biomarkers had a sensitivity of 100% (95% CI: 88%–100%) and a specificity of 100% (95% CI: 94%–100%) (Table 4). The AUC values for these five biomarkers were 1.000. An additional eight biomarkers (IL-8, CRP, resistin, thrombospondin, IL-1β, IL-6, IL-10, and IL-1α) demonstrated AUC values of greater than 0.9.

**Table 4:** Diagnostic characteristics of synovial fluid biomarkers

Biomarker	AUC	Cutoff	Specificity (%)	95% CI (%)	Sensitivity (%)	95% CI (%)
α-Defensin	1.000	4.8 μg/mL	100	95–100	100	88–100
ELA-2	1.000	2.0 μg/mL	100	95–100	100	88–100
BPI	1.000	2.2 μg/mL	100	95–100	100	88–100
NGAL	1.000	2.2 μg/mL	100	95–100	100	88–100
Lactoferrin	1.000	7.5 μg/mL	100	95–100	100	88–100
IL-8	0.992	6.5 ng/mL	95	87–99	100	87–100
SF CRP	0.987	12.2 mg/L	97	90–100	90	73–98
Resistin	0.983	340 ng/mL	100	95–100	97	82–99
Thrombospondin	0.974	1061 ng/mL	97	90–100	90	73–98
IL-1β	0.966	3.1 pg/mL	95	87–99	96	82–100
IL-6	0.950	2.3 ng/mL	97	89–100	89	71–98
IL-10	0.930	32.0 pg/mL	89	79–96	89	72–98
IL-1α	0.922	4.0 pg/mL	91	81–97	82	63–94
IL-17	0.892	3.1 pg/mL	99	92–100	82	63–94
G-CSF	0.859	15.4 pg/mL	92.	82–97	82	62–94
VEGF	0.850	2.3 ng/mL	77	65–87	75	55–89

AUC = area under the curve; α-defensin = human α-defensin 1-3; ELA-2 = neutrophil elastase 2; BPI = bactericidal/permeability-increasing protein; NGAL = neutrophil gelatinase-associated lipocalin; SF = synovial fluid; CRP = C-reactive protein; G-CSF = granulocyte colony-stimulating factor; VEGF = vascular endothelial growth factor.

**Fig. 1A–E:**
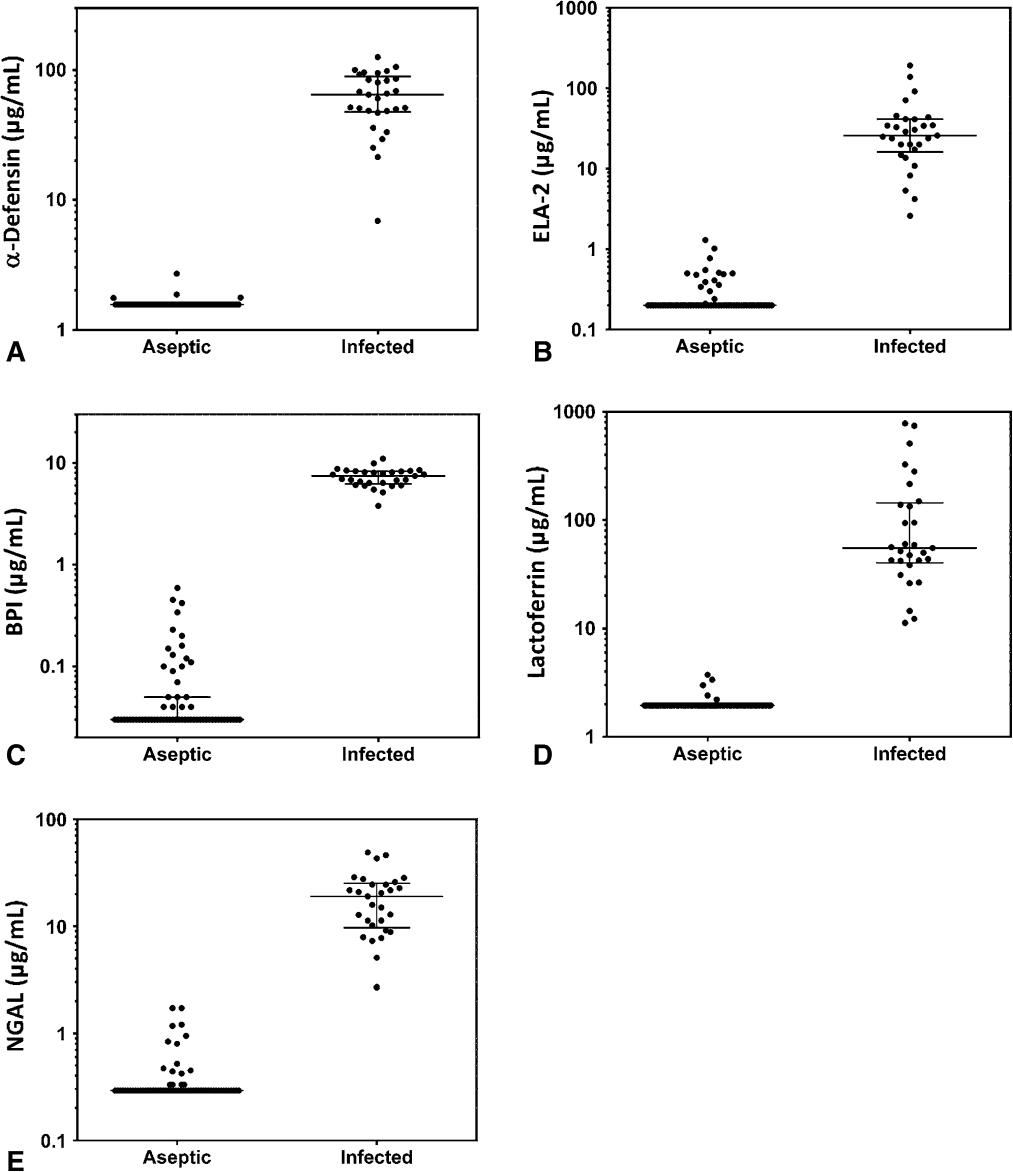
Log-scale dot plots demonstrate the diagnostic separation of study groups achieved by the five biomarkers achieving 100% sensitivity and specificity: (A) α-defensin, (B) ELA-2, (C) BPI, (D) lactoferrin, and (E) NGAL. The lowest reportable value was used for any samples that had a concentration below the limit of detection for each assay. Horizontal line = median; bars = interquartile range.

**Fig. 2A–D:**
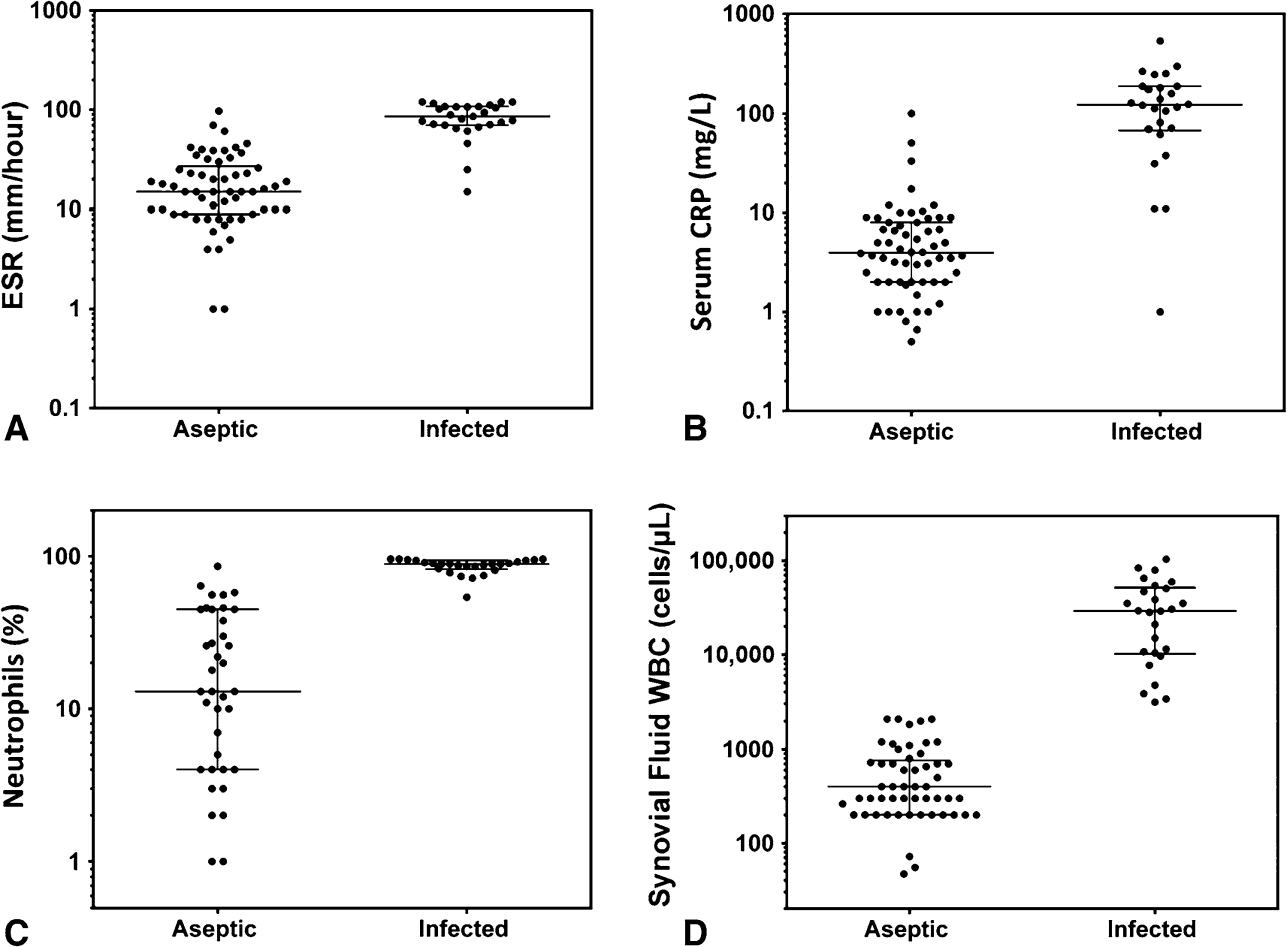
Log-scale dot plots demonstrate the diagnostic separation of study groups achieved by traditional tests for PJI: (A) ESR, (B) serum CRP, (C) neutrophil percentage, and (D) synovial fluid WBC count. Horizontal bar = median; error bars = interquartile range.

We further evaluated the five biomarkers demonstrating 100% sensitivity and specificity for PJI. Dot plots of these biomarkers compare the diagnostic separation of the aseptic and septic groups using median values and interquartile ranges (Fig. 1). For comparison, dot plots are also provided comparing the diagnostic separation of the aseptic and septic groups for ESR, serum CRP, synovial fluid WBC count, and neutrophil percentage (Fig. 2).

The five biomarkers demonstrating 100% sensitivity and specificity were compared to each other and to the synovial fluid WBC count using the Pearson correlation to evaluate for redundant performance among infected samples (Table 5). The mean correlation between biomarkers and the synovial fluid WBC count was 0.12 (range, −0.02 to 0.364), demonstrating no correlation. The biomarkers and synovial fluid WBC count had predominantly weak or no correlations with each other among samples with PJI.

**Table 5:** Pearson correlations with degree and type of correlation among patients with PJI

Biomarker	r value				
	α-Defensin	BPI	ELA-2	Lactoferrin	NGAL
α-Defensin					
BPI	0.40 (moderate +)				
ELA-2	0.25 (weak +)	0.22 (weak +)			
Lactoferrin	0.06 (none)	0.14 (none)	0.50 (moderate +)		
NGAL	0.23 (weak +)	0.44 (moderate +)	0.50 (moderate +)	0.75 (strong +)	
SF WBC count	0.08 (none)	−0.12 (none)	−0.02 (none)	0.31 (weak +)	0.36 (weak +)

PJI = periprosthetic joint infection; α-defensin = human α-defensin 1-3; BPI = bactericidal/permeability-increasing protein; ELA-2 = neutrophil elastase 2; NGAL = neutrophil gelatinase-associated lipocalin; SF = synovial fluid; WBC = white blood cell; + = positive.

There were no statistically significant differences between subgroups of patients in this study (systemic inflammatory disease versus other; antibiotic treatment versus other) in regard to mean ESR, CRP, synovial fluid WBC count, or biomarker.

## Discussion

The diagnosis of PJI has challenged surgeons since the advent of joint arthroplasty. There are several reasons for this diagnostic difficulty, including the absence of specific clinical signs and symptoms, the relative lack of accurate laboratory tests [20, 30, 32], and difficulties in culture isolation of pathogens due to prior therapy and formation of biofilms. The MSIS recently responded to this diagnostic difficulty by developing a definition for PJI [33]. As with any criteria-based tool, there are some practical clinical difficulties in using the MSIS definition for PJI, including (1) the subjective nature of several criteria, including the observation of purulence and interpretation of the frozen-section histology; (2) the delay in diagnosis required by waiting for several independent culture results; and (3) the relative complexity of the definition. A laboratory diagnostic test for PJI that provides a diagnosis matching the MSIS criteria would be highly desirable. Therefore, we evaluated the diagnostic characteristics of 16 promising synovial fluid biomarkers for PJI.

There are several weaknesses of this study. First, this study chose cutoff values to provide for the optimal performance of the biomarkers in this group of patients. Future studies may demonstrate a decline in performance when validating the cutoffs chosen in this study. Second, we excluded patients in the immediate postoperative period and those with suspected hip metallosis due to concerns that the MSIS criteria used in this study may not apply to these groups. Additionally, the subgroups of patients with cement spacers or systemic inflammatory disease were small. Therefore, it may not be valid to widely apply our results to these smaller subgroups until future studies with larger numbers are completed. Third, any diagnostic study is somewhat limited by the assumption that its patient population and prevalence of disease are similar to the more general population of such patients. The prevalence of PJI in this study was 31%, which is similar to the prevalence of PJI in a recent meta-analysis of 3909 patients tested for PJI (32.5%) [5]. In addition, we used sensitivity and specificity as the descriptive diagnostic measures in this study, which would not be affected by the prevalence of PJI in this study. As a final weakness, we included a heterogeneous group of patients in the aseptic disease group in an attempt to accurately represent the population of patients tested in clinical practice. These included patients with instability, patients with polyethylene wear, and patients with pain in the absence of an objective mechanical complication. The prevalence of diseases in the aseptic group could have hypothetically affected the specificity of our results. However, given the fact that many of the biomarkers exhibited 100% sensitivity and specificity for PJI, the decision to exclude patients with diagnoses other than aseptic loosening would not have substantially changed our results.

In this study, we evaluated 16 biomarkers for PJI based on nearly a decade of pursuit for optimal performance. Five biomarkers in the study provided a diagnosis that matched that of the MSIS definition for all 95 patients in this study, with 100% sensitivity and specificity. Eight additional biomarkers were identified with AUC values of greater than 0.9, exhibiting excellent diagnostic strength for PJI. Obviously, no test is perfect, and future studies with more patients, or those focusing on subgroups of patients, may demonstrate a decline in these results. Nevertheless, these biomarkers outperform historical reports of the currently used diagnostic tests for PJI, including serum CRP [2, 16, 20, 34], ESR [2, 16, 20, 34], and synovial fluid WBC count and differential [18, 35, 41], despite the inclusion of a more challenging group of patients.

The promise of synovial fluid biomarkers to diagnose PJI has been previously reported [8, 19, 22]. Similar to these previous studies, we found that cytokines and proteins with antimicrobial function provide the greatest utility for diagnosing PJI. To our knowledge, this study is the first to describe the performance of synovial fluid α-defensin, ELA-2, BPI, NGAL, and lactoferrin for the diagnosis of PJI. These biomarkers are all host proteins with direct antimicrobial activity, playing important roles in the innate response to eliminate pathogens [4, 11]. When pathogens are present, these biomarkers become more concentrated in the synovial fluid. Therefore, it is no surprise that these proteins are found to be diagnostically important for PJI.

If the biomarkers described in this study were merely mirroring the state of inflammation, we would have expected strong correlations among the biomarkers and between the biomarkers and synovial fluid WBC count. However, we did not identify many strong correlations between biomarkers and synovial fluid WBC count among infected patients. Nor did we identify many strong correlations among differing biomarkers. Therefore, it appears that these biomarkers are not merely redundant proxies for the local level of inflammation but instead are being modulated by other underlying causes.

There are several strengths of this study. First, to our knowledge, this is one of the largest diagnostic studies to date that utilizes the rigorous gold-standard MSIS definition for PJI. Second, patients usually excluded from similar diagnostic studies, such as those on antibiotics and those with systemic inflammatory disease, were included in this study to emulate standard clinical practice. In fact, 21% of infected patients in this study were on antibiotic treatment at the time of synovial fluid aspiration and 20% of all patients in the study had a history of systemic inflammatory disease, resulting in a historically challenging patient population. Finally, while some diagnostic biomarker studies limit their samples of PJI to a single organism [9, 19], our study included all infected patients diagnosed by the MSIS criteria, demonstrating utility of the biomarkers for most representative pathogens. Based on this study, which supports our earlier work [8, 9, 22], we conclude that synovial fluid biomarkers show promise as a valuable tool for the diagnosis of PJI. Given the ability of these assays to match the results of the more complex MSIS definition of PJI, we believe that these assays can improve the diagnostic accuracy in the field.
